# Somatic mutations and clonal evolution in normal tissues and cancer development

**DOI:** 10.1038/s12276-025-01592-0

**Published:** 2026-04-08

**Authors:** Kenichi Yoshida

**Affiliations:** Division of Cancer Evolution, National Cancer Center Research Institute, Tokyo, Japan

**Keywords:** Cancer genomics, Cancer genetics

## Abstract

Understanding the early stages of carcinogenesis requires detailed insight into the abnormalities present in normal cells before cancer onset. In the past, it was difficult to analyze genomic abnormalities in small clones in normal tissues. However, recent technological advances in genomic analysis have shed light on the process of accumulation of somatic mutations in normal cells, which is driven by factors such as aging and environmental influences. Even in normal tissues, clones that have acquired driver mutations—either directly contributing to carcinogenesis or adapting to specific pathological or genetic backgrounds—are frequently selected, leading to clonal expansion. Normal cells undergo clonal evolution into cancer cells over several decades, with the initial acquisition of a driver mutation occurring in early life. Here this review presents recent findings concerning the accumulation of somatic mutations in normal cells, acquisition of driver mutations and clonal evolution toward cancer.

## Somatic mutations drive clonal evolution in normal cells

Cancer often starts with genetic changes called somatic mutations, which build up in our cells as we age or due to environmental factors such as smoking. These mutations can lead to the growth of cell groups, or clones, that may eventually turn into cancer. This study explores how these mutations accumulate and how clones expand in normal tissues. Researchers studied small clones from various organs and analyzed genetic changes using advanced technologies such as laser-capture microdissection and next-generation sequencing. These approaches help understand how mutations accumulate in normal cells. The study found that certain mutations increase with age and are influenced by factors such as smoking and alcohol. Some driver mutations are common in cancer, while others are unique to normal tissues. The research highlights the importance of understanding these processes for early cancer detection and prevention.

This summary was initially drafted using artificial intelligence, then revised and fact-checked by the author.

## Introduction

Most cancers originate from genetic abnormalities, specifically the accumulation of somatic mutations. These mutations accumulate in normal cells in all organs of healthy individuals and are driven by factors such as aging, environmental influences and inflammation^1^. This often leads to the expansion of clones with driver mutations, thereby increasing tumorigenesis risk, ultimately progressing to precancerous lesions and cancer. The genetic background also plays a role in somatic mutation accumulation and clonal evolution. Furthermore, clonal evolution progresses owing to driver mutation acquisition, which may originate in the fetal state or at a young age. These findings provide an important foundation for understanding cancer development and is expected to contribute to the early detection and prevention of cancer in the future.

## Experimental techniques used to analyze clonal evolution in normal tissues

Clonal expansion in normal tissues is much smaller in size compared to tumor tissues. In organs other than blood, which is a liquid tissue circulating throughout the body, the genetic analysis of small clones is technically challenging^2^. Regarding clonal evolution in normal tissues, it was reported that the frequency of nonrandom X-chromosome inactivation increased with age in the blood of women^3^. Furthermore, data from single nucleotide polymorphism (SNP) array karyotyping, performed for genome-wide association studies, revealed the presence of mosaic chromosomal alterations (mCAs), including deletions, duplications and copy-neutral loss of heterozygosity (CN-LOH) in blood cells. mCAs increase with age and have been linked to hematologic cancer development^4,5^. Recent studies have shown that SNP array analysis detects low frequency chromosomal abnormalities (0.7–1%), and mCAs have been reported in 6–9% of healthy individuals^6–8^. Subsequently, genetic mutation analysis using next-generation sequencing revealed that clonal hematopoiesis, in which the acquired mutations may be a driver mutation common to hematologic tumors, also develops in healthy individuals and increases with age^9–12^.

**Fig. 1:**
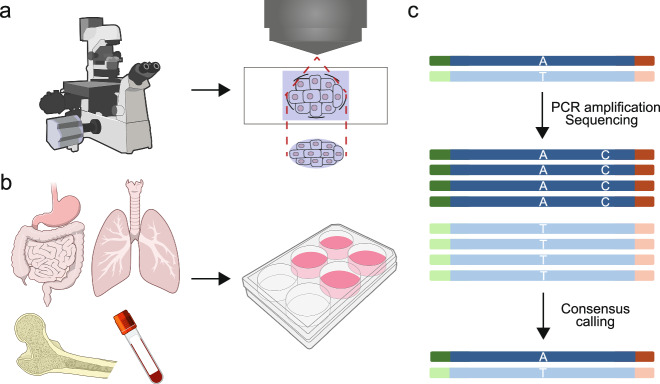
Methods to study clonal evolution in normal tissues. **a** By laser-capture microdissection, only the target type of normal cells is excised and analyzed. **b** Organoids or colonies derived from single cells of normal tissues from various organs are generated and analyzed. **c** In duplex sequencing, molecular barcodes are added to the double-stranded DNA, allowing for the distinction between errors and true mutations.

Sampling small clones in organs other than blood is challenging, and three methods are primarily used to study somatic mutations and clonal expansion in normal tissues. The first approach involves collecting relatively small samples for genetic analysis^13–15^. More recently, techniques such as laser-capture microdissection have enabled sampling of as few as 100–1000 cells (Fig. 1a) and genetic analysis using minute amounts of DNA for whole-genome sequencing has become feasible^16–24^. Although this sampling method enables the spatial analysis of clonal expansion, if the expansion is too small, the sample will be polyclonal, comprising a mixture of multiple clones. In such cases, the variant allele frequency of the somatic mutations may be low, making analysis difficult in many organs^25^. The second approach is to create and subsequently analyze organoids or colonies derived from single cells^23,26–32^ (Fig. 1b). This method is advantageous because genetic analysis is performed at the single-cell level, making it superior to the analysis of small samples. However, establishing single-cell-derived organoids or colonies requires substantial effort, and the creation of organoids is particularly challenging for some organs and diseases. Additionally, the lack of spatial information using this method makes clonal expansion analysis challenging. The third approach involves the use of recently developed technologies, such as duplex sequencing (Fig. 1c), which has a low frequency of sequencing errors and facilitates mutation analysis of bulk normal cells without clonal expansion^33,34^. For example, nanorate sequencing (NanoSeq) results in <5 × 10⁻⁹ errors per base pair (<30 errors in a diploid genome). This method can also be used for muscles and organs in the nervous system, where clonal expansion is less commonly observed because these cells do not undergo division, making microsampling-based techniques difficult^33^. Additionally, somatic mutations or copy number alterations have been detected using single-cell DNA analysis^35–38^ and RNA-sequencing analysis^39,40^.

## Mechanisms of somatic mutation accumulation in normal tissues

**Fig. 2:**
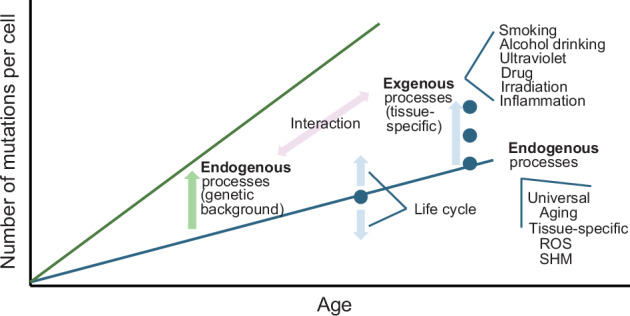
Accumulation of mutations due to endogenous and exogenous factors. With aging, mutations accumulate through universal or tissue-specific endogenous processes. Additionally, in several cancer predisposition syndromes, the accumulation of endogenous mutations is accelerated owing to genetic background. Moreover, the accumulation rate of endogenous mutations can vary depending on the life cycle. Furthermore, tissue-specific exogenous factors contribute to the accumulation of mutations, which may also interact with the genetic background. ROS reactive oxygen species, SHM somatic hypermutation.

Even in normal tissues before cancer onset, genomic abnormalities accumulate with age due to cellular divisions that begin with the first division of the fertilized egg, as well as endogenous processes independent of cell division (Fig. 2). A larger number of mutations (2.4–3.8 mutations per cell per cell division) are acquired during the initial division compared to later divisions (0.7–1.2 mutations per cell per cell division), and the daughter cells resulting from these divisions form tissues in an unequal manner^41–43^. Mutation signature analysis has been used to extensively study the process of somatic mutation acqusition^44^ in both cancer and normal cells, leading to many insights. Although the number of somatic mutations and the processes of accumulation may show slight variations among different organs (Table 1), endogenous mutation acquisition is a universal process that occurs in all organs^25^. Genomic abnormalities caused by various exogenous factors, including smoking, alcohol consumption, UV radiation, inflammation and medications, accumulate in the organs exposed to these factors. Additionally, genomic abnormalities are influenced by genetic background (Fig. 2). Individuals with congenital DNA mismatch repair deficiency have an increased predisposition to cancer and accumulate more somatic mutations than healthy individuals. Although chromosomal copy number abnormalities and structural abnormalities are relatively rare in normal cells, they increase with age in blood, breast tissue and other organs^4,37,40^. Environmental factors have also been linked to such abnormalities, including an association between increased Y chromosome abnormalities and smoking^45^. Some of the key processes involved in mutation acquisition in normal cells are discussed below (Table 2).

**Table 1:** Mutation burden, mutation signatures and driver mutations in each tissue.

	Number of SBSs per year	Signatures by endogenous processes	Signatures by exogenous processes*	Frequently mutated driver genes	Frequency of clonal expansion
Bladder	Unknown	Sig-B, SBS2/13	Sig-A (SBS92), Sig-C	*KMT2D*, *KDM6A*, *ARID1A*	High
Blood (Hematopoietic stem cell)	14.2–17	SBS1, SBS5, SBS_blood, SBS18, SBS2/13		*DNMT3A*, *TET2*, -Y	High
Blood (B lymphocyte)	15–17	SBS1, SBS_blood (SBS5-like), SBS8, SBS9	SBS7a		Low
Blood (T lymphocyte)	22–25	SBS1, SBS_blood (SBS5-like), SBS8	SBS7a		Low
Breast	19.5	SBS1, SBS5, SBS2/13		*PIK3CA*, der(1;16), +1q, del(16q)	High
Bronchus	22	SBS1, SBS5, SBS2/13	SBS4, SBS16, Sig-B	*NOTCH1*, *FAT1*, *TP53*	High
Colon	43.6	SBS1, SBS5, SBS18, SBS2/13	SBS88 (SBSA)		Low
Endometrium	29	SBS1, SBS5/40, SBS18		*PIK3CA*, *ARHGAP35*, *PIK3R1*	High
Esophagus	41.5	SBS1, SBS2/13, signature C	Signature D (SBS16)	*NOTCH1*, *TP53*, *FAT1*	High
Heart	19	SBS5 (signature A), SBS18 (signature C), SBS44 (signature D)			Low
Liver	33	SBS1, Sig-A, SBS9	SBS4, SBS22, SBS24		Low
Muscle	20.7	SBS1, SBS5-like (signature A, C)			Low
Neuron	17.1	SBS1, SBS5-like (signature A, C)			Low
Prostate	16	SBS1, SBS5			Low
Small intestine	42–51	SBS1, SBS5, SBS18, SBS2/13	SBS88		Low
Stomach	28	SBS1, SBS5/40, SBS18, APOBEC	SBS17, SBS28	*ARID1A*, *CTNNB1*, *KDM6A*	High

^*^The signatures caused by chemotherapeutic agents are excluded from the table.

**Table 2:** Mutational signatures detected in normal tissues and related mutational processes.

Signature	Tissues	Causes
SBS1	All tissues	Spontaneous deamination of 5-methylcytosine (clock-like signature)
SBS2/13	Blood, bladder, breast, colon, lung, skin, small intestine	Activity of APOBEC family of cytidine deaminases
SBS4	Lung, liver	Tobacco smoking
SBS5/40	All tissues	Unknown (clock-like signature)
SBS7(a/b/c/d)	Skin, lymphocyte	Ultraviolet light exposure
SBS8	Lymphocyte	Unknown
SBS9	B lymphocyte, liver	Polymerase eta somatic hypermutation activity
SBS10(a/b/c/d)	All tissues	Polymerase epsilon exonuclease domain mutations, defective POLD1 proofreading
SBS11	Blood	Temozolomide treatment
SBS12	Liver	Unknown
SBS16	Esophagus, liver, lung	Smoking, alcohol drinking
SBS17	Blood, colon, small intestine, stomach	Chemotherapy treatment (5-FU, capecitabine)
SBS18	Colon, endometrium, stomach*	ROS
SBS22(a/b)	Bladder, liver	Aristolochic acid exposure
SBS24	Liver	Aflatoxin
SBS25	Blood	Chemotherapy treatment (procarbazine)
SBS28	Colon, liver, stomach	Polymerase epsilon exonuclease domain mutations
SBS31	Blood	Platinum chemotherapy treatment
SBS32	Colon	Azathioprine treatment
SBS35	Small intestine	Platinum chemotherapy treatment
SBS36	Blood, colon	Defective DNA base excision repair due to *MUTYH* mutations
SBS41	Small intestine	Unknown
SBS44	Heart	Defective DNA mismatch repair
SBS88	Colon, small intestine	Colibactin exposure (*E. coli* bacteria carrying pks pathogenicity island)
SBS90	Blood	Duocarmycin exposure
SBS92	Bladder	Tobacco smoking
SBS99	Blood	Melphalan exposure

^*^Since SBS18 mutations can also be acquired in in vitro cultures, tissues in which SBS18 mutations were detected in such experiments were excluded from the table.

### Endogenous mutation processes

Increased age leads to increased accumulation of genomic abnormalities in the cells of all organs as the result of cell division and endogenous processes^25^, and the mechanisms, number of mutations and interacting factors are gradually being elucidated. The single-base substitution (SBS) signatures SBS1 and SBS5/40 have long been recognized as common mutation signatures universally present across all cancers^46,47^. These signatures reflect the endogenous mutation acquisition process and are considered clock-like because the number of mutations correlates with age^48^. The SBS1 signature is characterized by C>T mutations at CpG sites. It has been identified in most tissues, including nondividing neurons and muscles, and has been reported to increase with age, suggesting that the spontaneous deamination of 5-methylcytosine was fixed during cell division or fixed by the DNA repair process in nondividing cells^33^. The SBS1 signature has also been reported to occur during DNA replication by DNA polymerase ε^49^.

The SBS5 signature is relatively flat and characterized by a mild increase in C>T and T>C mutations, but its cause is unknown. Signature analysis of cancer and normal tissues has also revealed the presence of SBS40, which is similar to SBS5 (refs. ^25,47^). Both SBS5 and SBS40 mutations increase with age and have been identified in various organs, suggesting that they may be the same signatures^25^. However, SBS5 signatures exhibit slight pattern differences across different organs, implying that the mutations result from multiple mutagenic processes and show organ-specific diversity^21,33^. Mutations with a signature similar to SBS5 have been detected in blood^50,51^ and postmitotic organs, such as the brain and muscles^28,29,33^. These mutations increase with age, suggesting SBS5 accumulation via endogenous processes unrelated to cell division.

The SBS18 signature is characterized by C>A mutations and reflects DNA damage caused by reactive oxygen species (ROS)^52^. SBS18 has been identified in normal epithelial tissues in the colon, endometrium and gastric mucosa and increases with age^18,20^.

The SBS9 signature has been identified in normal memory B cells. This signature also increases with age and arises from error-prone DNA polymerase η bypass of background DNA lesions, somatic hypermutations (SHMs), induced by activation induced cytidine deaminase or oxidative stress-induced replication errors mediated in germinal center B cells^51^.

There are reports of various cancer predisposition syndromes characterized by genomic instability due to genetic background. In such cases, a larger number of somatic mutations accumulate in normal cells before cancer onset compared to cells from healthy individuals, resulting in an increased cancer risk. In cases with germline mutations in DNA polymerase ε or δ (a condition known as polymerase proofreading-associated polyposis), the incidence of colorectal and uterine cancer is high. Compared to normal individuals, the number of SBSs in intestinal crypts is approximately sevenfold higher in patients with *POLE* and up to threefold higher in patients with *POLD1* germline mutations. SBS signatures previously identified in cancers arising from these conditions have been determined as the cause, with SBS10(a/b) and SBS28 identified as responsible for *POLE* germline mutations and SBS10(c/d) responsible for *POLD1* germline mutations^53^. In contrast, in Lynch syndrome, which is caused by germline mutations in DNA mismatch repair genes such as *MLH1* and *MSH2*, the number of mutations in the colonic epithelial cells of patients was not increased compared to normal colonic epithelium^54^. This suggests that, despite the high incidence of colorectal cancer in Lynch syndrome, unlike *POLE* and *POLD1*, mutations in these genes do not impair DNA mismatch repair when only one allele is mutated. It is accepted that individuals with *BRCA1/2* germline mutations have a higher risk of cancer development, including breast cancer. In normal breast cells from *BRCA1/2* mutation carriers, the frequency of aneuploidy was reported as 3.63–3.65%, which was higher than the frequency of 2.45% reported in mutation-negative cases^38^.

Changes in the rates of mutation accumulation due to the aging process have been reported. In women, the number of accumulated mutations in normal breast epithelial cells is 19.5/year before menopause, decreasing to 8.1/year after menopause. Furthermore, a single pregnancy leads to a reduction of 54.8 mutations. These changes are the result of reduced cell division following menstrual cycle cessation and declined estrogen levels^23^.

### Exogenous mutation processes

One of the exogenous factors that induces somatic mutations is smoking. In cancers in organs directly exposed to tobacco smoke, such as lung and laryngeal cancer, the SBS4 signature, characterized by C>A mutations, was identified. Based on the results of experiments using induced pluripotent stem cells, SBS4 appears to be induced by benzo[a]pyrene, a polycyclic aromatic hydrocarbon that is one of the carcinogenic substances found in tobacco smoke^52^. Additionally, the SBS5 signature was reported to increase in cancers in organs that were not directly exposed to tobacco smoke^55^. Regarding the accumulation of genomic abnormalities in normal tissues due to smoking, an increase in SBS4 and SBS5 signatures was observed in normal bronchial epithelial cells derived from individuals with a smoking history^30,56^. Additionally, the SBS16 signature, previously reported in liver cancer associated with a smoking history^57^ and characterized by T>C transitions at ApTpN sites, was reported as increased in normal bronchial cells from donors with a smoking history. A novel mutation signature, Sig-B, has also been reported, which is similar to those induced by nitrosamines^58^, a group of carcinogens present in tobacco smoke, and similar to a mutation signature that has been observed in smoking-related head and neck cancers^59^. Furthermore, regarding organs not directly exposed to tobacco smoke, the SBS4 signature has been reported in the liver^19^, and a new mutation signature, SBS92 (Sig-A), was identified in the normal bladder^21^. Although smoking is a high risk factor for esophageal cancer, a specific smoking-related signature has not been identified in this organ^15^.

Alcohol consumption is associated with a high risk of various cancers, including esophageal cancer. An increase in the SBS16 signature (Sig. D), which is considered an alcohol-related mutation signature, was observed in esophageal epithelial cells of individuals with a history of alcohol consumption^15^. Furthermore, the rate of accumulation of SBS16 mutations was higher in individuals carrying the inactive variant of *ALDH2* (rs671), which is involved in acetaldehyde breakdown.

The SBS7 signature is caused by UV radiation and characterized by C>T mutations at cytosines preceded by another pyrimidine. It was initially identified in skin cancer and later found in epidermal cells, melanocytes and keratinocytes in the skin^13,31,60^. Skin cancer incidence varies according to ethnicity and higher frequencies occur in Western populations. Supporting this epidemiological difference, the mutation load associated with the SBS7 signature in skin keratinocytes in Asian populations was reported as lower than in Western populations, which was thought to be due to differences in genetic background^60^. Interestingly, SBS7 was also identified in acute lymphoblastic leukemia^61^ and normal B and T lymphocytes^51^, suggesting the acquisition of the SBS7 signature through UV exposure when the cells were present in the skin.

Certain strains of *Escherichia coli* in the gut microbiota cause DNA double-strand breaks (DSBs) in animal cells, leading to genotoxicity. Such bacteria are a potential risk factor for colorectal cancer development and typically harbor a large genomic gene cluster known as the pks island. The administration of pks-positive *E. coli* to colon organoids induced a characteristic mutation signature^62^ that matched the SBSA signature previously identified in normal colorectal crypts^18^. Thus, it was concluded that this signature is associated with pks-positive colorectal cancer (SBS88).

Secondary cancer development due to chemotherapy and the mechanism of action of anticancer drugs suggest that chemotherapy damages DNA in normal cells. The accumulation of mutations and mutation signatures in normal cells due to chemotherapeutic drugs was recently reported. Mutation signatures associated with alkylating agents have been reported in cancer for some time^46^, and mutations induced by chemotherapy even accumulate in normal cells. Mutation signatures caused by 5-FU (SBS17) and platinum (SBS35) were identified in normal colonic epithelial cells after chemotherapy^63^. Additionally, blood cell analysis after chemotherapy revealed mutation signatures induced by melphalan^64^ (SBS99) and other alkylating agents^65^. Furthermore, the antiviral drug ganciclovir, which acts as a competitive inhibitor of deoxyguanosine triphosphate incorporation into DNA, was reported to induce C>A mutations, contributing to secondary cancer development^66^. Similar accumulations of chemotherapy-induced mutations have also been reported in various tissues, other than the colon and blood^67^.

A mutation signature characterized by T>A mutations (SBS22) has been reported in hepatocellular carcinoma and bladder cancer associated with exposure to aristolochic acid, which is found in plants belonging to the *Aristolochia* genus. This compound causes kidney damage and has also been identified in hepatocytes and normal bladder epithelial cells^19,68^.

Ionizing radiation also causes the accumulation of genetic abnormalities^69^. Unlike other environmental factors that cause carcinogenesis, no characteristic SBS signature induced by irradiation has been identified. However, a new insertion–deletion mutation signature (ID-A) is thought to reflect the increase in deletions caused by random DSBs induced by irradiation. ID-A is partly characterized by an increase in deletions with microhomology and resembles the indel signature ID8, which is associated with nonhomologous end joining. Additionally, an increase in chromosomal structural abnormalities, which are relatively rarely associated with other factors, was observed. These abnormalities were characterized by breakpoints with microhomology, suggesting DSB repair via nonhomologous end joining. Besides balanced inversions, balanced translocations and long deletions, a low frequency of extrachromosomal DNA was also detected. Furthermore, complex chromosomal structural abnormalities, such as chromoplexy and chromothripsis, which result from ≥3 DSBs, were also observed^69^.

## Driver mutation acquisition in normal cells

**Fig. 3:**
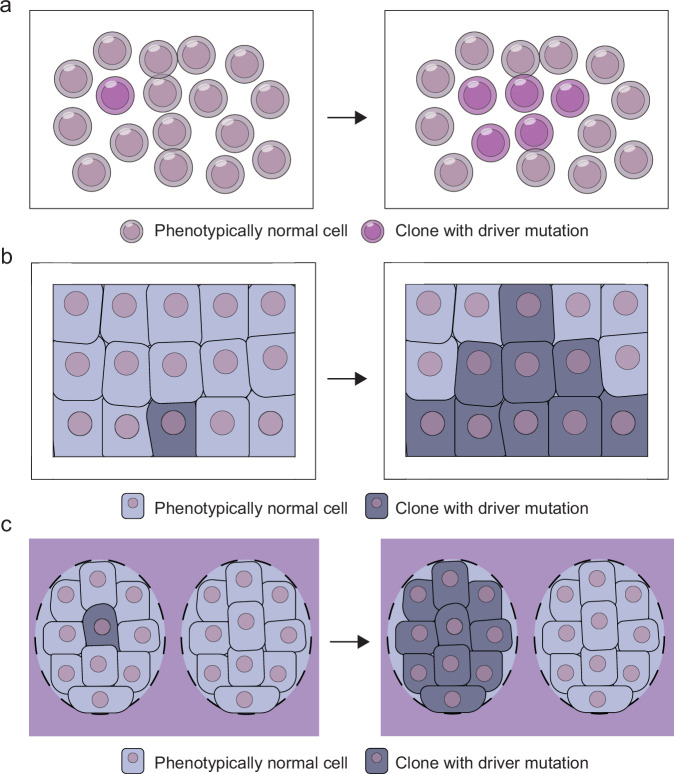
Clonal expansion due to the acquisition of driver mutations in normal tissues. **a** Since blood is a liquid organ, the clonal expansion of clones that have acquired driver mutations can grow in the blood circulating throughout the body and in the bone marrow. **b** In epithelial tissues, clonal expansion of clones that have acquired driver mutations is frequently observed with aging. **c** In organs such as the colon, prostate and liver, stem cells are localized in spatially isolated regions, making it difficult for clonal expansion to occur spatially even when clones with driver mutations emerge.

It has become clear that clones with specific genomic abnormalities are selected in normal tissues. These include driver gene mutations common to cancer or genomic abnormalities acquired in patterns different from those found in cancer. As explained in the previous section, clones that have acquired driver mutations emerge among the genomic abnormalities that accumulate owing to aging and environmental factors. These clones then further evolve, acquiring multiple driver mutations similar to those in cancer and leading to clonal expansion. SNP array analysis has detected the clonal expansion of blood cells with chromosomal abnormalities, such as del(20q), del(13q), del(11q), del(17p), trisomy 12 and trisomy 8, which are common in hematologic malignancies^4^. Furthermore, genomic analysis using next-generation sequencing has revealed that clones with acquired driver mutations common to myeloid tumors, such as *TET2* (ref. ^9^), *DNMT3A* and *ASXL1*, lead to clonal hematopoiesis (Fig. 3a)^10,11^, which has been reported as associated with the onset of age-related diseases^10,70,71^, such as cardiovascular disease and cerebrovascular disease. Conversely, clonal hematopoiesis has been linked to a decreased risk of Alzheimer disease^72^. High-sensitivity analyses have demonstrated the presence of clonal hematopoiesis in nearly all individuals aged >75 years^73,74^. However, it was also reported that the age at which clonal hematopoiesis is detected varies according to the affected genes, each of which has a different risk of progression to hematologic malignancies. For example, mutations in RNA splicing factor genes, such as *U2AF1*, *SRSF2* and *SF3B1*, which are common in older adults with myelodysplastic syndromes, are mainly observed in aged individuals and associated with a higher risk of progression to leukemia. In contrast, *DNMT3A* and *TET2* mutations, most frequently observed in clonal hematopoiesis, are acquired at relatively younger ages, and the risk of progression to leukemia is comparatively lower^75,76^. Moreover, genomic analysis of myeloproliferative neoplasms (MPNs) has revealed that driver mutations, such as those in *JAK2* and *DNMT3A*, are often acquired during early life, including the fetal state^77^.

Genomic analysis using small sampling techniques has revealed the spatial expansion of clones with acquired driver mutations in normal tissues (Fig. 3b). The expansion of clones with *NOTCH1* and *TP53* mutations is frequently observed in the squamous epithelium of the skin. Furthermore, acquired *NOTCH1* mutations were found in approximately 20% of skin cells, and the clones with acquired driver mutations were larger in size compared to clones without driver mutations^13^. The expansion of clones with acquired *NOTCH1* and *TP53* mutations has also been observed in esophageal cells. Tissue remodeling is driven by clones with driver mutations and progresses owing to aging and environmental factors, such as smoking and alcohol consumption^14,15^. Similarly, the selection of driver mutations has been observed in the epithelium of the main bronchi owing to aging and smoking^30^. As with blood cells, these driver mutations are often acquired during early life^15,30^. In the esophagus, *NOTCH1* mutations are acquired as early as infancy, and the expansion of small clones has even been observed in young, healthy individuals without carcinogenic risk factors such as as smoking or alcohol consumption^14,15^.

Driver mutations are frequently observed in the endometrium. Mutations in *KRAS* and *PIK3CA*, which are common in endometrial cancer, are acquired during childhood, before the age of 10 years^20^. Copy number alterations, including der(1;16), +1q and del(16q), which are commonly observed in breast cancer, as well as *PIK3CA* mutations, are often present in normal mammary epithelium and have been reported to occur from early puberty to late adolescence^23,37^. Mutations in chromatin remodeling genes, such as *KMT2D* and *KDM6A*, are frequently observed in the normal bladder. There are many cases of these driver mutations exhibiting convergent evolution, where clones within the same sample independently acquire different mutations in the same gene, suggesting that these mutations are strong drivers^21,68^. Similar to the bladder, mutations in chromatin remodeling genes, such as *ARID1A* and *KDM6A*, are frequently observed in normal gastric mucosal epithelium^24^.

There are some organs in which clonal expansion due to driver mutation acquisition is less likely to occur (Fig. 3c). For example, the frequency of driver mutations is relatively low (approximately 1%) in the normal colonic epithelium. This is because the colon is composed of units called crypts, which originate from a single stem cell, and even when driver mutations are acquired, they rarely lead to clonal expansion by disrupting the crypt structure^18,78^. Clonal expansion of acquired driver mutations in the normal prostate epithelium is also rare because the cells derived from stem cells are physically separated and located in distinct areas^22,79^. Similarly, the frequency of clones with acquired driver mutations is low (<5%) in the normal liver^19^.

There are some tissues in which clones acquire disease-specific driver mutations. In the blood, *PIGA* mutations and CN-LOH of chromosome 6p targeting the human leukocyte antigen region are frequently observed in aplastic anemia, in which pathogenic autoimmune mechanisms are involved^80,81^, and clones acquiring these abnormalities adapt through immune evasion. In chronic liver diseases, such as alcohol-related liver disease and nonalcoholic fatty liver disease, hepatocytes acquire mutations in metabolic-related genes, such as *FOXO1*, *CIDEM* and *GPAM1*, which may protect against lipotoxicity^82^. In the colonic epithelium of patients with inflammatory bowel diseases, such as Crohn’s disease and ulcerative colitis, clones with acquired mutations in genes related to the interleukin-17 signaling pathway, such as *NFKBIZ*, *TRAF3IP3* and *ZC3H12A*, are selected. These mutations help the cells evade damage caused by chronic inflammation^83–85^. Furthermore, mutations in these genes are relatively rare driver mutations in cancers that progress from their respective diseases, and they are considered to be unrelated to tumorigenesis or mutations that are negatively selected in the context of tumorigenesis^83^.

The acquisition of driver mutations and clonal evolution that adapts to the genetic background has been reported in congenital bone marrow failure syndromes, which are also cancer susceptibility syndromes^86^. Mutations in the *TERT* promoter and *POT1* occur in telomere biology disorders, such as dyskeratosis congenita, which compensate for telomere dysfunction^87^. Mutations in *EIF6* and del(20q), which involves *EIF6*, are present in Shwachman–Diamond syndrome and compensate for ribosomal maturation defects^88,89^. In diseases caused by recessive inheritance, such as Shwachman–Diamond syndrome, cases have been reported where clones with acquired CN-LOH or copy number alterations increase the number of relatively functional alleles from one of the two pathogenic alleles, thereby recovering function^90^. Such genetic alterations are known as adaptive rescue^86^. Conversely, clones with acquired *TP53* mutations evade apoptosis caused by TP53 activation induced by the dysfunction of telomeres or ribosomes and often progress to myeloid malignancies^87,88^, a process known as maladaptive rescue^86^.

## Clonal evolution in normal tissues and progression to precancerous lesions and cancer

Clonal hematopoiesis increases the risk of hematologic malignancies. Myeloid clonal hematopoiesis is associated with myeloid tumor development (hazard ratio of 7.0), and lymphoid clonal hematopoiesis is linked to lymphoid tumor development (hazard ratio of 4.2)^91^. It is estimated that 0.5–1% of individuals with detectable clonal hematopoiesis develop hematologic malignancies each year^10^. Furthermore, an analysis of longitudinally collected samples revealed that clonal expansion occurs gradually over 5–10 years, eventually leading to the development of hematologic malignancies^75,76^. Moreover, MPN analysis revealed that the average time from the acquisition of the initial driver mutation, such as *JAK2* V617F, to MPN onset was approximately 30 years, taking several decades for the acquisition of a second driver mutation that would lead to tumorigenesis^77,92^.

Phylogenetic analysis of normal breast epithelial cells, precancerous lesions, and cancer from the same case suggested that following the acquisition of der(1;16) during early puberty to late adolescence, it took over 10 years for the development of breast cancer through the acquisition of further driver mutations^23^. Notably, multiple independent cancer lesions arose from a common noncancerous clone carrying der(1;16), demonstrating a pattern different from the traditional model of cancers evolving from a single cancer founder.

Differences between clones in normal tissues and precancerous lesions or cancers have been investigated. In the bladder, liver and bronchi, the number of mutations and copy number alterations in clones in normal cells was reported as substantially lower compared to cancer in the same individual^19,30,68^. Conversely, there was no consistent differences in the number or types of driver mutations between clones in normal breast tissue and cancer in the same individual, suggesting the contribution of epigenomic changes to phenotypic differences^23^. Although details of the phenotypic changes that occur in normal cells following the acquisition of early driver mutations remain unclear, the acquisition of driver mutations in hematopoietic stem cells increases the clonal expansion rate^74,77^.

Some of the driver mutations observed with clonal expansion occur frequently in normal tissues but relatively infrequently in cancer, such as *NOTCH1* mutations in the skin, esophagus and bronchial tissues. However, the role of clones with such mutations requires further elucidation. *Notch1* mutations have been reported to promote the proliferation of normal esophageal epithelial cells in mouse models while inhibiting the proliferation of cancer cells, suggesting that mutant *Notch1* clones may play a role in tumorigenesis suppression^93^. In contrast, several typical driver genes in cancers, such as *TP53* in various tissues^14,15,20,21,30^, *FGFR3* in bladder^21^ and *PTEN* in endometrium^20^, occur at lower frequencies in normal tissues compared to cancers.

Undoubtedly, the genetic background influences the process of clonal evolution from normal cells to cancer. Individuals carrying an SNP in the *TCL1A* promoter region showed suppressed expansion of clonal hematopoiesis driven by mutations in *TET2* and *ASXL1*. The presence of this SNP inhibits the expansion of hematopoietic stem cell clones through the expression of TCL1A induced by these driver mutations^94^.

## Summary

This review has outlined the accumulation of somatic mutations in normal cells, the acquisition of driver mutations and subsequent clonal evolution caused by various factors. Although substantial progress has been achieved over the past decade, many aspects remain poorly understood, such as the impact of the genetic background and the interaction between the genetic background and environmental factors. For example, the co-existence of *Helicobacter pylori* infection and germline *BRCA1*/*2* mutations has been shown to increase the risk of gastric cancer^95^. In clonal hematopoiesis, clonal expansion often occurs without driver mutation acquisition^74^. However changes in the epigenome (epi-mutations) in normal tissues, which represent one of the nongenomic abnormalities involved, require further clarification^74^. Although the understanding of epi-mutations in normal cells is limited, these changes may partly explain the clonal evolution driven by abnormalities other than those in the genome, as seen in cancer^96^.

The mechanism of driver gene mutation-induced changes in cellular phenotypes and the reason why only a limited number of clonal expansions in normal tissues evolve into cancer despite the presence of numerous clones with driver mutations remain unclear. Additionally, an increase in clones with few tobacco-related genetic mutations has been observed in normal bronchial epithelium after smoking cessation^30^. Thus, the mechanism of clonal evolution or turnover that occurs in response to environmental changes remains unclear. It is expected that progress will be made in clarifying these aspects, resulting in a deeper understanding of carcinogenesis and ultimately contributing to early cancer detection and prevention.
